# Zebrafish: an emerging real-time model system to study Alzheimer’s disease and neurospecific drug discovery

**DOI:** 10.1038/s41420-018-0109-7

**Published:** 2018-10-03

**Authors:** Suraiya Saleem, Rajaretinam Rajesh Kannan

**Affiliations:** Neuroscience Lab, Molecular and Nanomedicine Research Unit, Centre for Nanoscience and Nanotechnology, School of Bio and Chemical Engineering, Sathyabama Institute of Science and Technology, (Deemed to be University), Jeppiaar Nagar, Rajiv Gandhi Salai, Chennai, 600119 Tamil Nadu India

**Keywords:** Alzheimer's disease, Neuroscience

## Abstract

Zebrafish (*Danio rerio*) is emerging as an increasingly successful model for translational research on human neurological disorders. In this review, we appraise the high degree of neurological and behavioural resemblance of zebrafish with humans. It is highly validated as a powerful vertebrate model for investigating human neurodegenerative diseases. The neuroanatomic and neurochemical pathways of zebrafish brain exhibit a profound resemblance with the human brain. Physiological, emotional and social behavioural pattern similarities between them have also been well established. Interestingly, zebrafish models have been used successfully to simulate the pathology of Alzheimer’s disease (AD) as well as Tauopathy. Their relatively simple nervous system and the optical transparency of the embryos permit real-time neurological imaging. Here, we further elaborate on the use of recent real-time imaging techniques to obtain vital insights into the neurodegeneration that occurs in AD. Zebrafish is adeptly suitable for Ca^2+^ imaging, which provides a better understanding of neuronal activity and axonal dystrophy in a non-invasive manner. Three-dimensional imaging in zebrafish is a rapidly evolving technique, which allows the visualisation of the whole organism for an elaborate in vivo functional and neurophysiological analysis in disease condition. Suitability to high-throughput screening and similarity with humans makes zebrafish an excellent model for screening neurospecific compounds. Thus, the zebrafish model can be pivotal in bridging the gap from the bench to the bedside. This fish is becoming an increasingly successful model to understand AD with further scope for investigation in neurodevelopment and neurodegeneration, which promises exciting research opportunities in the future.

## Facts

- Zebrafish can be used as a model to study various disease pathologies in Alzheimer’s disease.
- Laser axotomy coupled with time-lapse imaging and 3D imaging reveal interesting facts about degeneration/regeneration in Zebrafish larvae.
- New approaches to treat Alzheimer’s disease can be further unearthed with this model.

## Open questions

- How does zebrafish serve as a model for understanding Alzheimer’s disease?
- Can real-time imaging in zebrafish address the major breakthroughs in the field of Alzheimer’s research?
- Can zebrafish merge the gap between neurospecific drug discovery and clinical research?

## Introduction: zebrafish is emerging as a robust model for study of human neurodegeneration

**Fig. 1:**
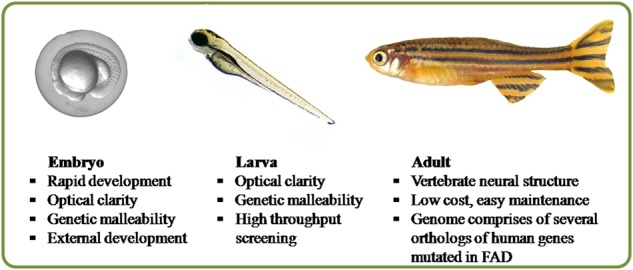
Advantages of using zebrafish as an AD model

The zebrafish (*Danio rerio*) is a prominent vertebrate model system for comprehensive analysis of the unique functions of genes along with their signalling pathways during development and neurodegeneration^1^. Such studies have been possible as the zebrafish possesses several distinct advantages over other vertebrate models (Fig. 1).

**Fig. 2:**
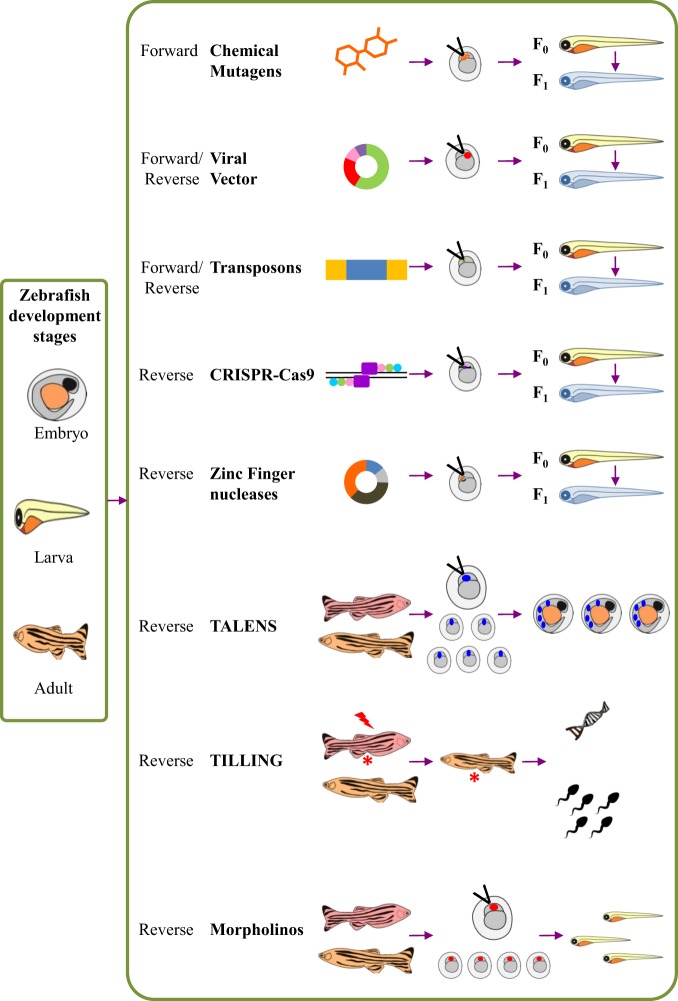
Possible genetic manipulations in zebrafish: ‘forward genetic’ tools exist to create random mutations in the zebrafish genome. Several ‘reverse genetic’ methods are also in use to identify and characterise zebrafish genes of interest either by overexpression or by knocking it out. For inducing mutations researchers have used chemical mutagens like Ethyl-Nitroso Urea, viral vectors, transposon-based mutagenesis by conditional “gene trapping” and “gene breaking”, zinc finger nucleases, and the more recent one being, clustered regularly interspaced short palindromic repeats (CRISPR-Cas9) systems, which provide the advantage of gene manipulation with ease and high efficiency, eliminating any unwanted off-target effect. Morpholinos, based on the antisense oligonucleotide gene knockdown technique, are the most regularly used reverse genetic tool for gene manipulation in zebrafish. They are designed in such a way that they bind to specific locations on the transcripts from genes of interest. The mode of action of morpholino can be by either blocking translation or interfering with the proper splicing of the exons. ‘Targeting Induced Local Lesions in Genomes’ (TILLING) method, has also been successfully adapted to zebrafish. Most recently the Transcription Activator-Like Effector Nucleases (TALENS) system is the most potent system for targeting genes in the zebrafish model

- Owing to the simplicity of their natural habitat, it is much easier to maintain them in a laboratory than to simulate the conditions essential for mammals. Thus, zebrafish can be grown in a cost-effective manner. Their short generation times of 3–5 months enhances the rate of experimental progress^2^.
- They possess external fertilisation and their development pattern facilitates the observation and experimental manipulation of the embryos. Moreover, they have large clutch size varying between 200 and 300 per fish, which ensures a ready supply of animals for research work.
- One of the most unique advantages of the zebrafish is the unrivalled optical clarity of the embryos, allowing visualisation of individual genes (fluorescently labelled or dyed) throughout the developmental process using non-invasive imaging techniques^3–8^. This transparency of the embryo also helps in genetic manipulations.
- Owing to the small size of the larvae, high-throughput screening of neuroactive compounds can be easily performed.
- It is very easy to introduce transient manipulation of gene activities and their subsequent examination in a normal cellular environment^9,10^ (Fig. 2). The embryos are quite malleable to genetic manipulation by morpholino antisense oligonucleotide, mRNAs, transgenes and genome editing techniques like CRISPR-Cas9, TALENS^11–13^.
- The zebrafish possess a vertebrate neural structural organisation and their genome has several gene orthologs similar to those mutated in human Familial Alzheimer’s disease (FAD). Very recently in an interesting study, scientists have upgraded the web tool for analysing zebrafish genes using gene ontology as the entire zebrafish genome has been sequenced^14^.

Therefore, zebrafish pose a better model system than rodents as they allow in vivo analysis without disturbing the physiological milieu of the disease.

## AD and pathogenesis

Alzheimer’s disease (AD) is a progressive neurodegenerative disease. It is the most prevalent form of dementia in the world affecting almost 47 million people worldwide (Alzheimer’s Association)^15^. The number of Alzheimer’s patients is projected to reach 82 million globally by 2030, and the number is expected to rise to 152 million by 2050 of which Asia-Pacific alone shall contribute 71 million cases. The total cost for AD is estimated to be $183 billion, which might rise up to $1.1 trillion by 2050^16^. The two main pathological hallmarks of the disease include Aβ plaques and neurofibrillary tangles^17^. Other clinical features include depression, hallucinations, speech impairment, motor disabilities and aggressive behaviour^18^. Though extensive research has been done, yet, early diagnosis of AD is still not possible^19^. The latter stages of AD include significant neuronal loss in specific regions of the brain, ultimately leading to shrinkage of the total volume of the brain^20–22^. AD can be categorised into two main types, familial AD (FAD) and sporadic AD (SAD)^23^.

FAD shows an autosomal dominant inheritance and is usually caused by mutations occurring mainly in three genes, Amyloid precursor protein (APP), Presenilin 1 (PSEN1) and Presenilin 2 (PSEN 2)^23,24^. FAD accounts for only ~ 1–5% cases of AD, whereas the rest is attributed by SAD^25^. SAD is attributed mainly by a combination of environmental risk factors and genetic susceptibility. Reports suggest a functional role of Apolipoprotein E (ApoE) phenotype in the late-onset AD^26^, ε4 allele being the major risk factor for AD, whereas ε2 allele is protective^27,28^.

The presence of Aβ plaques in the brain led to the origin of the Aβ hypothesis^29^. It suggests the pivotal role of Aβ in initiating and triggering the pathology of the disease including inflammation and oxidative stress^30^. For decades, Aβ deposition and aggregation have been treated as the primary mechanisms underlying the disease pathology. Though Aβ hypothesis is central to the disease, scientists, however, have propounded several other hypotheses^31,32^. Among them, the earliest proposed was the cholinergic hypothesis.^33^ Decrease in the cholinergic transmission in AD is responsible for the abnormalities in the cognitive and functional domains of AD patients. However, it is now clear, that cholinergic dysfunction can cause cognitive impairment only through indirect mode^33^. Another most prevalent hypothesis is the tangles hypothesis.^34^ A better correlation exists between presence of tangles and status of the disease progression; however, the amyloid hypothesis holds as it has been noticed that mutations in Tau do not result in plaque deposition. Among other hypotheses, the calcium hypothesis explains how the activation of the amyloidogenic pathway regulates the calcium signalling in the neurons to affect cognition^35,36^. However, the relation between dysregulated calcium signalling, aging and cognitive decline are still not clear^36^. The mitochondrial hypothesis suggests that the mitochondria plays a major role for the production of reactive oxygen species and subsequent neurodegeneration^37^. The complete link between mitochondria and the disease is yet to be established. Hence, a better understanding of the various hypotheses in AD will definitely direct our approach for better treatment strategies of the disease.

## Zebrafish as an AD model

### Neuroanatomical similarity

Research shows a high conservation between zebrafish and human brain organisation^38^. A great degree of similarity between their neuroanatomic^39–41^ and neurochemical pathways^42^ are reported. The medial, dorsal and lateral pallium of the zebrafish are similar to the amygdala^43^, isocortex^44^ and hippocampus^45,46^ in other vertebrates, respectively. The zebrafish encephalon comprises of the forebrain, mid brain and hind brain, (diencephalon, telencephalon and cerebellum). Similar to mammalian brain, the zebrafish brain harbours the main excitatory glutamatergic and inhibitory GABAergic neurotransmitter circuits^47^ along with the presence of muscarine cholinergic receptors^48^. Besides, they possess GABA, glutamate, serotonin, dopamine, histamine, acetylcholine neurotransmitters^49^, enzymes of synthesis and for metabolism^50,51^. Similarity exists even at the cellular level, as the cell types astrocytes^52^, microglia^53^, oligodendrocytes^54^, cerebellar Purkinje cells^55^, myelin^56^ and motor neurons^57^ are found similar to human cells. Further studies on the neuronal patterns in the spinal cord of adult zebrafish, neural differentiation and development of spinal network establish their similarity to higher order vertebrates^58–60^.

### Behavioural similarity

Several anthropomorphic assays have been performed to depict similar behavioural pattern between zebrafish and humans, which implicates a conserved behavioural mechanism and circuitry paradigm of both systems. Researchers have employed the zebrafish behavioural pattern to study physiological behaviour like feeding^61–63^, learning^64^, hearing^65^, vision^66^, touch^67–69^ and emotions like fear^70–72^, pain^73^, helplessness^74^, courtship^75^, social interactions^69,76,77^, anxiety^78^ and decision making^79^. Further, reports indicate similarity between expression patterns and axonal projections of hypocretin/orexin neurons of larval zebrafish and humans^80^. Interestingly, because of the high degree of behavioural similarity between zebrafish and humans; researchers have also employed the use of circadian rhythm in zebrafish locomotor activity to understand the mechanism, which can regulate sleep in humans^80–83^. Sleep behaviour pattern has been studied in the zebrafish and has been simulated with sleep-like state in mammals. This study shows that the rest phase in zebrafish and the behavioural manifestations of sleep in mammals exhibit considerable fundamental similarities. They further report that the zebrafish exercise homoeostatic control over rest behaviour which is regulated by the circadian rhythm, features similar to that of mammals^84^. Disruptions in the circadian rhythm in AD have been reported by several groups^85–90^. Although a link between circadian cycle and AD is clearly implicated, a proper mechanistic explanation connecting them is still lacking. The interactions among the proteins involved in maintaining circadian rhythm in the zebrafish is quite similar to those observed in the mammals^91–94^. Apart from this, the cholinergic neurotransmitter system modulates drug induced reward activity in the zebrafish making it compatible to study the neurobiology of addiction. This establishes the zebrafish as a robust model for studying the biology of behaviour in vertebrates^95^.

### Pathophysiological resemblance

Several zebrafish models have been established by placing Aβ central to the disease pathology to simulate AD. A group of researchers suggest that higher levels of Aβ monomers can stimulate angiogenic sprouting in the developing zebrafish hind brain^96^. Quite interestingly, another group found both APP and Aβ-deficient larvae displayed cerebrovascular defects. Interestingly, these anomalies could be reversed by treating the embryos with human Aβ_1–42_ peptide. However, there was no effect when they were treated with p3 (the shorter APP cleavage product)^97^. This finding brought to light the significant cerebrovascular growth promoting function of Aβ. Further, the embryos treated with 2.5 µM of Aβ_1–40_ led to abnormal vasculature development and cell death^98^.

Besides the Aβ model, research has also focussed on the generation of zebrafish models with Tauopathy^99^. Cytoskeletal disruption occurred on expressing frontotemporal dementia with Parkinsonism linked to chromosome 17, a mutant form of human Tau in the neurons of the zebrafish, which resembled the neurofibrillary tangles observed in AD^100,101^. Another group expressed mutated form of the human τ protein in the zebrafish neurons which disrupted the cytoskeletal structure^101^. Although removal of exon2 from Enolase GFP-transgenic zebrafish and its replacement with complementary DNA that encoded for the four-repeat isoform of the human Tau resulted in eight times overexpression of Tau in the zebrafish brain compared with normal human brain. The overexpressed Tau localised to axons and resembled neurofibrillary tangles^102^. These reports exemplify the use of zebrafish as a model for AD.

### Zebrafish as a neuropharmacological model

Researchers have checked non-associative learning in the zebrafish larvae based on cognitive and behavioural responses of AD. Seven days post fertilisation (dpf) larvae were exposed to a series of acoustic stimuli, to which the larvae displayed significant reduction of startle response^103^. Another group injected Aβ_1-42_ into the hind brain ventricle of 24 h post fertilisation (hpf) zebrafish embryos. They observed significant cognitive deficits in the embryos with increased Tau phosphorylation in target residues of GSK-3β in the 5 dpf larvae^104^. Zebrafish embryos exposed to Trimethyltin chloride (TMT) exhibited neurobehavioural toxicity, specifically, apoptosis in the tail, modulations in photomotor response and frequency of tail flexion^105^. Most recently, another pharmacologic model of AD has been developed by adding okadaic acid^106^. Both Aβ plaques and phosphorylarion of Tau have been found to increase with increasing concentrations of okadaic acid. Learning and memory deficits have also been observed in these fish^106^. Zebrafish treated with Aluminium in an acid environment displayed behaviour with AD-like condition wherein their locomotor activity and learning ability got abrogated^107^. Furthermore, it has been reported that intraventricular injection of Aβ_1–42_ in the embryonic brain leads to memory loss and cognitive deficits along with increased Tau phosphorylation^104,108^. The zebrafish is therefore emerging as a powerful model for research in the field of neuropharmacology.

### Zebrafish as neurogenetic model

Researchers have unravelled the role of APP in FAD using the zebrafish model, whereby, APP knockdown zebrafish displayed defective convergent-extension movements with reduced body length and short curled tail. They showed that Swedish mutant APP is unable to overcome the developmental defects unlike the wild type APP^109,110^. In addition, it has been shown that the Sortilin-Related Receptor (SORL1)-dependent switch can divert APP from the late endosomal pathway. This sequesters the APP into the endosomes, thus preventing the formation of Aβ^111,112^. Another group has showed that the wild type Psen1 in the zebrafish facilitates anomalous Aβ_1–42_ secretion similar to mutations associated with FAD^113^. Zebrafish embryos injected with morpholinos, which block Psen1 translation are viable but show p53-dependent apoptotic death of neurons^114–117^. A unique function of the Psen1 has come to light through studies in a mutant zebrafish lacking Psen1 activity. These mutant fish are viable and reveal the regulation of histaminergic neuronal development by Psen1 in the fish^118^. Unlike Psen1, loss of Psen2 expression hinders the synthesis of dorsal longitudinal ascending interneurons from the spinal cord during the development of zebrafish larvae^119^. In an interesting study, the scientists report the pathological role of a new truncated isoform, PS2V of Psen2 and its implication in neurodegenerative diseases^117^. Research has focussed on the β secretase as well, whereas Bace1 and Bace2 knockout zebrafish were generated using zinc finger nuclease-mediated genome editing. Bace1 mutants showed hypomyelination in the peripheral nervous system while Bace2 mutants showed anomalous migration of the melanocytes^120^. However, the Bace1/2 double knockout zebrafish did not display any enhancement in the mutant phenotypes, pointing out to the non redundant function of Bace1 and Bace2. Another Aβ toxicity model was generated by using the mitfa (nacre) gene promoter for the expression of the Aβ gene. The gene was chosen as it could produce a distinct disrupted pigmentation pattern in the larvae. However, the model did not turn out to be successful as the differential pigmentation pattern was evident in the adult fish, only at 16 months, by which time, the fish were infertile and too old for breeding^121^. Fluorescently labelled Tau transgenic zebrafish model of AD with a mutation Tau-P301L were also generated, which presented with the key pathological features of Tauopathy including neurofibrillary tangles, neuronal loss and cell death^122^. The zebrafish therefore harbour a huge resource of genetic information, which need to be manipulated to reveal the molecular details of AD.

## Disadvantages of zebrafish as an AD model

There are several advantages in using zebrafish as a model system for studying AD and a few limitations in using them in translational neuroscience research. Pharmacological modifications in the fish are easily brought about by adding the desired chemicals in water. However, the quantification of chemical compound entering the fish is unpredictable, as substances can be absorbed randomly through the gills and skin of the fish owing to exposure of the whole body in the aqua medium^123^. Further, the fact that zebrafish-specific Aβ peptide is yet to be elaborated remains a drawback. More research also needs to be performed to study whether the post translational processing of APP in humans is also prevalent in the zebrafish^124^.

Zebrafish possesses a unique ability of regenerating neurons along their rostrocaudal brain axis throughout life unlike mammals. In an interesting observation, the researchers found that zebrafish microinjected with Aβ_1–42_ peptide showed regeneration of neurons, specifically neural cell/progenitor cell proliferation and neurogenesis^125^. They studied the regenerative ability in old and young fish to understand the influence of aging and Aβ deposition on neuroregeneration. They show that in neurodegeneration induced by Aβ, microglia get activated, to prevent synaptic degeneration and promote neurogenesis. Thus they establish a potential link between neurodegeneration, neuroinflammation and neurogenesis^125^. This might pose a threat to the feasibility of the AD model but it definitely opens up a whole new world of research to delve into the molecular mechanisms of signalling pathways that could be active in playing a paramount role in the regeneration of neurons. This will surely pave the way for understanding the molecular programmes required for regeneration of the mammalian central nervous system.

## Real-time neurological imaging studies in zebrafish

The zebrafish has a relatively simple nervous system, which allows imaging of neurons easily. This creates the possibility to visualise specific neuronal proteins of interest and thus provide a wonderful opportunity to study the neurological processes in detail^49,126–130^. Zebrafish larvae are small transparent vertebrates, which are highly suitable to confocal microscopy when labelled with dyes^131^. Thus, it facilitates studies regarding the in vivo physiological signalling processes in intact organ systems^132^.

Real-time imaging using zebrafish has had a profound impact on the understanding of major physiological processes such as neurodegeneration (Table 1). Researchers could detect early pathological features like hyperphosphorylation and conformational changes of Tau within the first 2 days of embryonic development by real-time imaging using a stable transgenic zebrafish^122^. After a few days, the larvae developed substantial neurodegeneration displaying all pathological features including neurofibrillary tangles by 5 weeks of development. The pathological features develop much earlier in zebrafish as compared with the other available rodent models. As the zebrafish model provides the opportunity for easy manipulation and visualisation of the optically clear embryos^122^. Based on this, real-time imaging of microglial phagocytosis has enhanced the perception of microglia mediated neurodegeneration^53^. A group of scientists used laser imaging technique to measure the activity of nerve cells in zebrafish, propounding a novel association between Neurexin2 and spinal muscular atrophy; establishing neurexin2 as a potential target for the treatment of SMA^133^. In vivo fragmentation of mitochondria was observed by real-time imaging when exposed to apoptosis-inducing agents in a mitochondrially targeted GFP-transgenic zebrafish^134^. Another group studied the effect of mitochondrial oxidation on vulnerability to axonal degeneration by time-lapse confocal imaging^135^. Detailed real-time imaging analysis of the timing of cell division in zebrafish embryo has also been analysed by real-time imaging^136^. Recently developed techniques like laser axotomy coupled with time-lapse imaging have revealed the role of extrinsic cell types in degeneration and regeneration in the zebrafish larvae^137^. Real-time imaging using zebrafish has therefore unearthed a vast amount of information in AD, whereas the potential exists for a lot more to be brought to light.

**Table 1:** Alzheimer’s disease: insights revealed by real-time imaging in Zebrafish

Paper	Findings	References (Pubmed ID)
Plucinska G et al. 2012	Microtubule-affinity regulating kinase 2 (MARK-2), regulates axonal transport in a Tau-dependent manner.	23152604
Kim MJ et al. 2008	In vivo fragmentation of mitochondria upon exposure to the following apoptosis-inducing drugs: valinomycin, carbonyl cyanide 4-(trifluoromethoxy) phenylhydrazone (FCCP) and staurosporine.	18778258
Leung LC et al. 2013	Imaging zebrafish neural circuitry.	23630470
Renninger SL et al. 2013	Two-photon calcium imaging of neural population activity in zebrafish.	23727462
Feierstein CE et al. 2015	Functional mapping of circuits in behaving zebrafish.	25433239
Ahrens MB et al. 2012	Brain-wide dynamics at single-cell resolution using two-photon calcium imaging during behaviour.	22622571
Aizenberg M et al. 2011	Conditioned stimulus and unconditioned stimulus activate different subsets of neurons in the cerebellum, using calcium imaging in zebrafish.	21677154
Panula P et al. 2006	Analysis of modulatory neurotransmitter systems and behaviour in zebrafish.	18248264
Moritz C et al. 2015	σ1 receptor modulates microglial responses in neurodegeneration.	25666889
Casano AM et al. 2016	Role for developmental apoptosis in the long-term positioning of microglia in the zebrafish brain.	27425604
Paquet D et al. 2009	Neuronal cell death induced by TAU was imaged by time-lapse microscopy in vivo.	19363289
Peri F et al. 2008	a1 subunit mediates fusion between phagosomes and lysosomes during phagocytosis.	18510934
Kozawa S et al. 2016	In vivo imaging and real-time prediction of cell division timing in developing zebrafish embryo.	27597656
Ritter DA et al. 2001	Predictions regarding varied behavioural roles for different classes of spinal interneurons.	11698606
Gahtan E et al. 2002	Widespread distribution of neural control systems in the zebrafish brain.	11784774
Takahashi M et al.	In vivo imaging of functional networks inhibitory in nature on the mauthner cell of larval zebrafish.	12019312
Creton R et al. 2000	Requirement of apo-aequorin during embryonic development for calcium imaging?	9502193
Higashijima S et al. 2003	Use of cameleon for imaging neurons.	12930818
Muto A et al. 2016	Functional imaging of the brain under natural behavioural conditions.	27464819

### Ca^2+^ imaging in zebrafish

Neuronal development of the larva and adult has been studied using the calcium imaging technique. These studies have presented us with a better understanding of the basic biological principles during development and adulthood of vertebrates^138^. Bulk loading approach involves the use of synthetic Ca^2+^ indicators to label cells within the tissue. This technique has been utilised to study the reticulospinal neurons or Mauthner cells in the larval zebrafish^139,140^. Microinjection of a chemical dye, Oregon Green BAPTA-1 results in specific labelling of neuronal cell bodies in the zebrafish brain^140^. Another group of researchers injected the calcium green dextran to study the Mauthner cell circuit in the living fish^139^. The next improvisation in this field is the use of aequorin, a bioluminescent photoprotein that emits light upon binding to Ca^2+^^141^. Aequorins have been successfully used for Ca^2+^ imaging in the zebrafish^142,143^. The subsequent development in this field is the use of in vivo two-photon Ca^2+^ imaging. Understanding of the functional topology of neural activity patterns has been possible using this technique^144,145^. Another recent development in this area is the use of genetically encoded calcium indicators such as cameleon. Their major advantage is the ability to monitor neuron activity in a non-invasive manner in the living zebrafish^146^. Calcium imaging has also provided insights into the cellular events that execute axonal dystrophy arising in neurodegeneration^147,148^. Thus the Ca^2+^ imaging technique has helped in understanding the functional role of neurons which would not have been possible by other approaches.

### 3D imaging in zebrafish

The zebrafish is yet again adept for one of the most recent imaging techniques, which is the three-dimensional reconstruction of images to produce a 3D volumetric representation of the specimen. It includes three-dimensional time-lapse imaging of embryos either by confocal or multiphoton laser scanning microscopy. This technique employs the use of varied signals for analysis of the image, including two-photon-excited fluorescence, second harmonic generation and third harmonic generation^149^. Selective plane illumination microscopy, also known as light sheet fluorescence microscopy is another technique used in 3D imaging^150^. Apart from this, researchers have also used fluorescent probes to construct 3D imaging in live zebrafish^151^. This serves for high-throughput imaging ensuring sufficient reproducibility of observations. Vertebrate automated screening technology has been used to take multiple axial views of the zebrafish larvae. These were later reconstructed to produce 3D volumetric representation of the larvae and also its measurements, thus providing a more holistic view^152^. Extensive automated processing techniques have also been developed to analyse the complex images for easier comprehension^153^. 3D high resolution imaging is a rapidly developing technique but is accompanied by some technical issues like high expense, normalisation, artefact corrections and web-based publication of results among others. A recent discovery has addressed one such issue by coming up with a multi-usage observation chamber called UniverSlide adapted for live 3D bio imaging of the zebrafish larvae^154^.

3D imaging in zebrafish is a most recent technique and still rapidly evolving, which allows the visualisation of the whole organism, thus leading to an elaborate understanding of the in vivo scenario in the organism. It is therefore a highly promising technique that could produce a breakthrough in the field of zebrafish imaging and thus help it to stand out as a star model for future research.

## Neurospecific drug discovery using zebrafish model

Several approaches have been made to effectuate the discovery of potential therapeutic compounds exploiting the optical clarity of the zebrafish embryos and larvae. Owing to the small size of the embryo and larvae, drug discovery in a high-throughput scale can be achieved using this model system^155^. Till date, very few studies involving neurological drug discovery have been performed in the zebrafish, most of the studies being performed outside the nervous system^156–160^. Evaluation of a number of psychoactive compounds have also been performed using zebrafish^161^. Although it has been found that both nicotine^162^ and ethanol^163^ affect the development of the nervous system, cocaine^164,165^, amphetamine^95^, morphine^166,167^, ethanol^168,169^ and nicotine have been noted to elicit reward or anxiety-related behaviour in the zebrafish. Interestingly, nicotine has been reported to have cognition enhancing effects in a learning paradigm in zebrafish^170^.

A group of researchers identified a small molecule called prostaglandin E2, which regulates hematopoietic stem cell homoeostasis^171^. Small molecules that could relieve cell cycle arrest^172^ and cardiovascular problems^173^ and also modulate the embryonic heart rate^174^ by suppressing mutations have also been screened using zebrafish model system. Most importantly, research shows that the effect of these drugs is similar in humans and in zebrafish^158,175^, thus validating that zebrafish models for drug discovery have the potential to produce or identify therapeutic compounds suiting human conditions^176,177^.

Studies have also been performed to analyse the effect of several neuroactive compounds in the zebrafish adults, whereby addition of such compounds in the water of the fish tanks have been shown to differentially change their swimming pattern and diving behaviour. Several neuroactive compounds like piracetam, methylenedioxymethamphetamine, hallucinogenic agents like mescaline and phencyclidine, a nitroamine explosive 1,3,5-Trinitroperhydro-1,3,5-triazine, kynurenic acid, Δ^9^-tetrahydrocannabinol and heroin (diacetylmorphine) have been tested using zebrafish^178–184^. Rapid behaviour-based screening has also evolved as an inexpensive assay for identifying small neuroactive molecules^130,185^.

The blood–brain barrier (BBB) of the zebrafish has molecular and functional similarity to the higher vertebrates^186–188^. Analysis performed using transmission electron microscopy, fluorescent markers, chromatography and tandem mass spectrometry have revealed that BBB in the zebrafish is both structurally and functionally similar to that of mammals. Its development starts by 3 dpf and continues upto 10 dpf in the zebrafish larvae^189^. Another group reports the presence of tight junctions in the BBB as the brain endothelial cells show immunoreactivity to Claudin-5 and Zona Occludens-1^190^. Recently a group of researchers suggested an orthotopic glioblastoma model of zebrafish, which could be used as an efficient assay system for visualising the BBB penetrating efficiency of anti-GBM drugs^191^. Given the feasibility of high-throughput screening and the advantage of similarity with mammals, it is reasonable to say that zebrafish offers an excellent opportunity for screening neurospecific compounds.

## Controversies around the use of zebrafish for research on AD

Aβ plaques are most widely believed to be pivotal to the pathogenesis of AD^32,192,193^. They lead to synaptic dysfunction, disruption of neuronal connectivity and neuron death^194–199^. Interestingly, research in the zebrafish model of AD suggests that Aβ may also play a role in maintaining cerebrovascular functions^97^. They show that Aβ deficiency leads to reduction in cerebrovascular branching and vessel length in the developing hind brain of zebrafish embryo. In corroboration to this, another group of researchers reported that Aβ is involved in regulation of angiogenesis in the human umbilical cord vein and in the zebrafish hind brain^96^. This role of Aβ in the zebrafish stands in complete refutation to its functionality in the humans; wherein it causes cerebrovascular dysfunction, leading to cognitive defects^200,201^. One probable reason for this discrepancy could be the fact that Aβ in the teleosts is different from the other vertebrate Aβ including humans^97^. The group further explains why drugs targeting Aβ production failed in recent clinical trials as the potential function of Aβ in regulating angiogenesis could be interfering with the mechanism of action of the drug. Further research on the molecular mechanism of this functionality shall help understand the discrepancy better^92^. This also points out to the existence of other non-amyloid hypotheses (cholinergic hypothesis^33^, tangles hypothesis^34^, calcium hypothesis^35^ and mitochondrial hypothesis^37^ among others) in AD. Several zebrafish AD models have already been established with focus on Tauopathy^102,122^ and cholinergic hypothesis^202^ while better models exhibiting a combination of several pathologies still remain to be studied. Elaborate studies involving real-time imaging in the zebrafish model may unravel potential roles of these hypotheses in AD pathogenesis. With these controversies, zebrafish emerges to be even more interesting as a model system for neurodegeneration which needs further research. It also holds promise as a complete model for understanding AD and providing a platform for research on areas which have not been dealt with earlier but could prove to be beneficial in the field of drug development.

## Future of zebrafish as a model for AD

In the present review, we aim to illustrate the eminent progress that zebrafish propounds in order to comprehend the pathological mechanisms of AD. It can be envisaged that zebrafish model has emerged as an interesting tool for strategic study of AD^203^. The gap between drug discovery based on cellular models and pre clinical assays can be efficiently bridged by research using this model system. The zebrafish presents itself as the best candidate for high-throughput pharmacological screening of drugs before validating them in rodent models. Abundant research has already been performed to elaborate on the scope of zebrafish as a model to understand AD. However, arenas like the behaviour, physiology, neuroanatomical circuitry of the fish and the link between neurodevelopment and neurodegeneration (Table 2) still need to be better elucidated. A good AD transgenic model expressing both Aβ and Tau pathologies is the current requirement. This will help in completing the puzzle of understanding AD, of which some pieces are still lacking. We conclude that the zebrafish has already come a long way as a potential model in the field of neurodegeneration, whereas it continues to emerge as an attractive model system for future research in AD. It definitely possesses a vast potential to be capitalised upon for developing therapeutic interventions for AD.

**Table 2:** Link between neurodevelopment and neurodegeneration: scope for research in zebrafish

Paper	Findings	Scope of research	References (Pubmed ID)
Liu N et al. 2012	mir-34 regulates healthy aging and long-term brain integrity in *Drosophila*. mir-34 mediates its function by silencing its target E74A gene, which is required for brain development but is harmful during aging.	Exploring this micro RNA may provide an insight into the aging process and disease susceptibility.	22343898
Boehm M et al. 2005	microRNA *lin-4* and its target, the putative transcription factor *lin-14*, control the timing of larval development and also regulate life span in the adult *Caenorhabditis elegans*.	To study the role of homologues of this microRNA to understand processes responsible for determining life span in vertebrates.	16373574
Huang X et al. 2011	In *C. elegans*, Zinc finger protein SEA-2 regulates larval developmental timing by controlling the expression of heterochronic gene lin-28. SEA-2 also regulates aging in a DAF-16/FOXO-dependent manner.	To identify the subset of genes that regulate aging in adults and development in larval stages.	21471153
Glynn P et al. 2000	Neuropathy target esterase (NTE) is an integral membrane protein in vertebrate neurons maintains interactions between neurons and glial cells during development. Whereas it leads to disrupted calcium signalling and elevated Calpain activity in neurodegeneration condition.	To explore the mechanism by which NTE maintains communication between neurons and glia to help understand these interactions in the vertebrate system.	10759065
